# Work-related stressors and coping behaviors among leaders in small and medium-sized IT and technological services enterprises

**DOI:** 10.1186/s12889-023-15581-3

**Published:** 2023-04-14

**Authors:** Indra Dannheim, Anette E. Buyken, Anja Kroke

**Affiliations:** 1 Regional Innovative Centre of Health and Quality of Live Fulda (RIGL), Fulda University of Applied Sciences, Fulda, Germany; 2 Department of Nutritional, Food and Consumer Sciences, Fulda University of Applied Sciences, Fulda, Germany; 3 Institute of Nutrition, Consumption and Health, Faculty of Natural Sciences, Paderborn University, Paderborn, Germany

**Keywords:** Work-related stressors, Coping Behavior, Leadership, SMEs, Health-oriented leadership, Occupational health

## Abstract

**Background:**

Occupational health interventions for leaders are underrepresented in small and medium-sized enterprises (SMEs). When creating and developing effective occupational health interventions, identification of the specific needs of the target group is regarded as an essential step before planning an intervention. Therefore, the aim of this study was (1) to examine the subjectively experienced work-related stressors of leaders in small and medium-sized IT and technological services enterprises, (2) to explore coping behaviors leaders use to deal with the experienced work-related stressors, (3) to investigate resources supporting the coping process and (4) to identify potentially self-perceived consequences resulting from the experienced stressors.

**Methods:**

Ten semi-structured interviews with leaders in small and medium-sized IT and technological services enterprises were conducted. The interviews were transcribed and analyzed with content-structuring qualitative content analysis in accordance to Kuckartz.

**Results:**

Leaders in small and medium-sized IT and technological services enterprises experience various stressors caused by work organization as well as industry-related stressors and other work-related stressors. To address the experienced stressors, leaders apply problem focused coping behaviors (e.g. performing changes on structural and personal level), emotional focused coping behaviors (e.g. balancing activities, cognitive restructuring) as well as the utilization of social support. Helpful resources for the coping process include organizational, social and personal resources. As a result of the experienced work-related stressors, interviewees stated to experience different health impairments, negative effects on work quality as well as neglect of leisure activities and lack of time for family and friends.

**Conclusion:**

The identified experienced work-related stressors, applied coping behaviors, utilized resources and emerging consequences underpin the urgent need for the development and performance of health-oriented leadership interventions for leaders in small and medium- sized IT and technological services. The results of this study can be used when designing a target-oriented intervention for the examined target group.

**Supplementary Information:**

The online version contains supplementary material available at 10.1186/s12889-023-15581-3.

## Background

The prevention of work-related stress and psychosocial risks has gained importance due to their significant impact on individuals, organizations and national economies [1–3]. Target-oriented and effective interventions hold the potential to reduce both social and economic costs of health impairments [4–6]. The analysis of work-related stressors, including those of leadership staff, is therefore of high importance for the development of effective occupational health interventions [7].

Although these health interventions foster and promote individual health and well-being in worksite settings, occupational health interventions in small and medium-sized enterprises (SMEs; < 249 employees and < 50 million yearly sales [8]) are underrepresented [9]. An examination on the application of workplace health promotion among 825 enterprises in Germany revealed that 20% of SMEs do not implement occupational health interventions. In contrast, only 1.8% of large enterprises had not established a workplace health promotion program [10]. When SMEs reported health promotion activities these were often focused on legal requirements, such as occupational safety regulations rather than offering diverse occupational health interventions [10, 11]. In addition, occupational health interventions addressing leaders were rarely found in SMEs. When asked about future occupational health activities, large scale enterprises acknowledged the importance of leadership competences as a central capacity for being able to cope with work-related stressors more frequently than SMEs [10].

When creating and developing effective occupational health interventions, using elaborate planning tools such as the Intervention Mapping Approach, identification of the essential needs of the target group is regarded as an essential step before planning an intervention [12].

A specific but not well recognized target group are leaders in small and medium-sized information technology (IT) and technological services enterprises – due to an increased application of IT and related technological services [13] a vital and rising industry [14, 15]. In the past, research primarily reported results on employees working in IT and technological services including leaders rather than analyzing leaders in SMEs separately [16–20]. Although available results provide insight into industry-specific working conditions, coping behaviors and resulting consequences, evidence on work-related stressors and their consequences, coping behaviors and resources supporting the coping process is limited and almost no respective information is available from leaders working in small and medium-sized IT and technological services enterprises. Additionally, past research does not adequately reflect changes in recent years. The COVID-19-pandemic amplified and still amplifies the digitalization process in worksite settings [21]. These changes, especially the resulting high market demand, led to increasing and changing leadership challenges in these enterprises [22], many of which are SMEs [23]. As a consequence, the development and performance of health-oriented leadership interventions in IT and technological services companies gain increasing importance. Therefore, the aim of this study was (1) to examine the subjectively experienced work-related stressors of leaders in small and medium-sized IT and technological services enterprises, (2) to explore coping behaviors leaders use to deal with the experienced work-related stressors, (3) to investigate resources supporting the coping process, and (4) to identify self-perceived consequences resulting from the experienced. The results of the study serve as a first step in the development of targeted, effective health-oriented leadership interventions in the context of IT and technological services enterprises.

## Methods

### Theoretical considerations

As a first step in the (qualitative) research process, a theoretical framework is to be defined, and the understanding of terms and concepts is to be explained. Since this research activity focusses on work-related stress, respective stress models were selected. For this study, the transactional stress model by Lazarus and Folkmann, and an occupational psychological extension by Bamberg et al. were chosen [24, 25]. This approach was selected because it describes the development of stress and enables to derive preventive activities for reducing work-related stress and psychosocial risks [24, 26]. According to the transactional stress model, stress arises when situations are perceived as harmful or threating [24]. This appraisal process is, according to Lazarus and Folkmann, divided into three phases. In the primary appraisal it is decided whether the situation is perceived as irrelevant, positive/favorable or harmful/threating. If the situation is perceived as harmful/threating, the stress process is revealed. The secondary appraisal involves an assessment of available resources and coping strategies [24]. Primary and secondary appraisal do not strictly follow each other in time, but can overlap and influence each other. After the first two appraisal processes, individuals apply coping behaviors. In a third step, depending on its coping success a potentially reappraisal of the situation is performed. Coping is described as “cognitive and behavioral efforts to manage specific external and internal demands that are appraised as taxing or exceeding the resources of the person.” [24]. Lazarus and Folkmann differentiate between problem and emotional focused coping. Problem focused coping refers to actions directed at solving or removing a problem, emotional focused coping refers to situations aimed at regulating one´s emotion [24, 27]. Bamberg et al. criticized that external factors influencing the stress process were not sufficiently considered in the transactional stress model. Consequently, they extended the model incorporating the elements stressors, resources and consequences [25]. Stressors are described as external factors of stress situations [25, 28]. Work-related stressors refer to characteristics of the work situation and can be seen as risk factors for experiencing stress [25, 28]. Resources mitigate, reduce or avoid the occurrence of stressors and have a direct effect on the way individuals cope with experienced stressors [25, 29]. Consequences of stress emerge both short and long term, and affect somatic health, cognitive function, emotions and behavior. These consequences influence not only individuals themselves, but also family and friends as well as working environments [25] (see result section for illustration of applied theoretical approach).

### Study design

To examine work-related stressors coping behaviors, helpful resources and consequences resulting from the experienced stressors among leaders in small and medium-sized IT and technological services enterprises, a qualitative approach was used as this allows to explore complex human issues by providing a comprehensive and in-depth analysis of the contextualized account of personal experiences [30]. In total, ten semi-structured interviews were conducted by using the problem-centered interview (PCI) method according to Witzel. The PCI method allows to capture individual experiences, specific behaviors and subjective perceptions of interviewed persons, and aims to forge a consistent understanding between interviewee and interviewer [31]. To ensure the quality of reporting on the methodology of this qualitative study, the COREQ (Consolidated criteria for reporting qualitative research) checklist (checklist contained in additional file 1) was applied [32].

### Interview guideline

An interview guideline was developed to address the formulated research aims. As outlined by Witzel, the guideline enables to structure the interview and assures to cover all relevant topics and comparability of data material [31]. The interview guideline containing multiple narrating questions was divided into the topics: work and work-related stressors, coping behaviors, resources and consequences. Prior to the interviews, the interview guideline was tested with three leaders working in different occupational sectors. Minor changes in the order of the questions asked had to be made.

### Data collection

Data were collected in June and July 2021. Participants were recruited via professional and social contacts of the first author as well as the snowball system. The study sample comprised ten German-speaking leaders working in the field of IT and technological services in companies with less than 250 employees who had more than three years’ experience in their leadership position. In this study, leaders are defined as persons who are authorized to give instructions to at least one employee and who are held responsible for that employee’s actions and work.

The study was conducted in a non-commercial, publically funded research and transfer project. All interviews were conducted by the first author, a female health scientist serving as researcher in the field of occupational health, who had prior experiences in conduction and interpretation of qualitative interviews. Enterprises of six interviewees were cooperation partners in this funded research and transfer project. Prior relationships to all interviewees did not exist or only referred to seeing them, but not directly talking to them, on events of the publically funded research and transfer project. All leaders participated voluntarily, with informed consent and without reimbursement. The interviews lasted between 30 and 55 min (mean = 42.8 min). Three interviews were performed online via video call and six interviews were conducted in workplace premises of the interviewees. Detailed information on the participants is available in Table 1 (see result section). According to the recommendations of Creswell [33], and in order to create a comfortable conversation, it was ensured that the location was quiet and free from distraction. Prior to recording, interviewees were informed that they could reject answering any question and withdraw from the interview without reason if they feel uncomfortable at any time. Further, all participants were asked to sign a consent form regarding the performance and recording of the interview. Postscripts were created directly after the interviews by the first author. To protect the identity of the participants, identification codes (ID 1–10) were used and results are presented in masculine form.

### Data analysis

Interview recordings were transcribed professionally by external transcribers following the transcription rules of Claussen et al. [34]. Transcripts were analyzed in their original language to preserve original meaning. Only the quotes used were translated to English.

In this study, content-structuring qualitative content analysis in accordance to Kuckartz [35] was used for data analysis. This approach enables a systematic, rule- and theoretical analysis of the data material towards the goal to develop a category system representing the data material. In a first step, categories were derived deductively according to the research aims and based on theoretical and model-guided presumptions. Text passages, which referred to the main categories, were assigned to the categories and extracted. In a second step, subcategories were formed inductively within the main categories based on the available data. The category system was developed by working through the whole data material revising, modifying and summarizing the encoded categories. Encoding was performed by the first author using the software application MAXQDA® 2018. Interviewees were neither involved in the process of data transcription nor in the analysis. Additional file 2 displays the identified category systems including selected quotes representing each category.

## Results

### Study sample

The study sample consisted of nine males and one female working in leadership positions in small or medium-sized IT and technological services enterprises, with seven of them being the owners. Participant´s age ranged from 34 to 62 (mean = 43.8 years). All participants lived in a relationship. The working years in the leadership position ranged from three to forty-three years. The number of managed employees varied between 8 and 80, with an average of 30 employees. Further information on the sample is displayed in Table 1.

**Table 1:** Study participants general characteristics (n = 10)

Gender	Age* (years)	Professional background	Enterprise owner	Years of leadership experience*	Number of managed employees*	Children
Male	30–39	Technical	Yes	3–10	20–30	Yes
Male	30–39	Technical	Yes	3–10	20–30	Yes
Male	30–39	Business	Yes	3–10	30–40	No
Female	30–39	Social	Yes	10–20	20–30	Yes
Male	30–39	Technical	Yes	10–20	20–30	Yes
Male	40–49	Technical	No	10–20	0–10	No
Male	40–49	Technical	No	10–20	10–20	No
Male	50–60	Technical	No	10–20	> 80	Yes
Male	50–60	Business	Yes	20–30	20–30	Yes
Male	> 60	Technical	Yes	> 40	30–40	Yes

* For data protection reasons only ranges are displayed

**Fig. 1:**
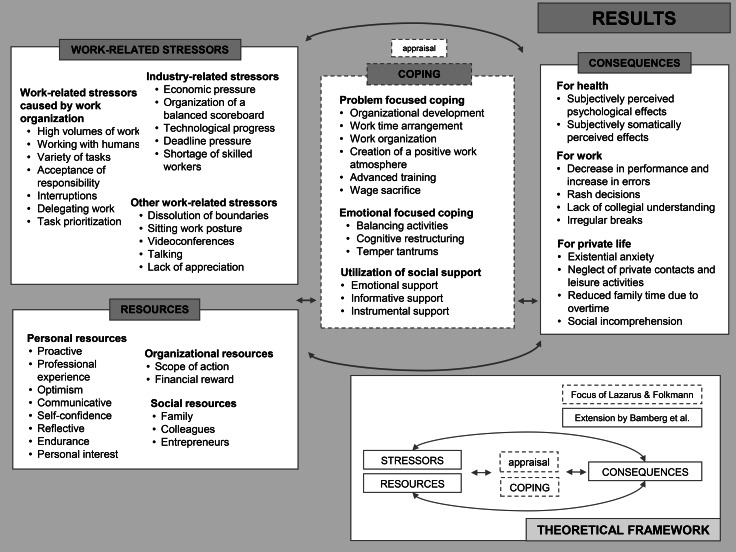
Summary of results (own demonstration) and illustration of the applied underlying framework (own demonstration after Bamberg et al. [25])

In the following, the identified categories and subcategories including selected quotes to underline the results will be presented separately. Figure 1 provides a summary of the identified categories.

### Work-related stressors

In the interview material, three major categories regarding work-related stressors could be identified: (1) work-related stressors caused by work organization, (2) industry-related work stressors, and (3) other work-related stressors.

#### Work-related stressors caused by work organization

The most frequently mentioned stressor by all interviewees was *high volumes of work*. Working weeks with an average of 60 to 80 h were described as normal. All interviewees reported *working with humans* as being stressful. Specifically, working with different target groups, e.g. employees, colleagues, clients and customers, who have varying occupational backgrounds, experiences, demands and interests, was described as source of stress. One interviewee described to feel like a psychologist in some moments:

*“(I: What would you say is the biggest challenge for you as a leader?) The 30 employees. Yes, well, of course I always say that for fun, but it’s just really like that, you’re just a bit of a psychologist.” [ID 9]*

In addition, most interviewees described having a wide *variety of tasks*: Not only organizational and strategic tasks related to their managing position but also project work is experienced as challenging:

*“The biggest challenge is certainly to accommodate a number of things, the actual business, the actual daily project work with then, of course, the superordinate and also, to have notion of the projects, and also to acquire projects.” [ID 10]*

Many interviewees named the *acceptance of responsibility* as an experienced work-related stressor. It was mentioned that having responsibility for company success and employees results in taking risks, making unpleasant decisions and not being able to hide behind some else:

*“As a leader, you are an escalation manager, as the saying goes. Basically, you have to deal with things that don’t work most of the time. Yes. And that takes its toll on you. And you have to make sure, in some way, that you can still sleep.” [ID 6]*

Next to the burdening factor of *interruptions*, which occur due to telephone, email or employee requests, some interviewees expressed difficulties in *delegating work* and *task prioritization*.

#### Industry-related work stressors

A major industry-related work stressor identified is *economic pressure.* The interviewees reported that economic pressure arises from their entrepreneur and leadership position being responsible for economic success as well as demands of clients and customers.

The majority of interviewees mentioned that the *organization of a balanced workload* is experienced as stressful, as unexpected requests from consumers and clients would make it challenging to provide balancing workloads for employees and themselves. Further, *technological progress* was identified as industry-specific stressor according to the statements of the interviewees. Leaders explained that new technologies constantly arise requiring continuous development.

Next to *deadline pressure*, interviewees also indicated the *shortage of skilled workers* as major industry-specific stressor. One interviewee described that his main task refers to finding skilled workers:

*“Our focus is very clear, we want to attract skilled workers to our company, because everyone is having these problems in my industry right now – we can’t get people. So basically, that’s my main job right now.” [ID 9]*

#### Other work-related stressors

A major other work-related stressor identified was *dissolution of boundaries* as interviewees reported having problems detaching from work. Reasons for the dissolution of boundaries were reported to arise from incidents such as employees contacting leaders late in the evening, answering emails or taking phone calls during vacations. One interviewee reported that his leadership position led to changes in behavior which is then applied in private life as well:

*“But that [the leadership position] also changes the character of oneself, you must not forget that either. Because it’s hard to shake off this leadership role. That means that you also apply this at home with friends and children. That’s automatically the case. I always have discussions with my wife when we talk. She then says, “Yes, now you’re coming with your executive topics. This is not a staff meeting here.“ I mean, you don´t want to turn a private conversation into a staff meeting, but you just notice that the leadership position is in you.” [ID 7]*

Some interviewees experienced *sitting work posture*, *videoconferences* or constant *talking* as burdening factor. Also a *lack of appreciation* was mentioned when asked about experienced work-related stressors.

### Coping behaviors

With respect to study aim 2 (exploration of coping behaviors used to deal with experienced work-related stressors), three coping behaviors could be identified: (1) problem focused coping, (2) emotional focused coping, (3) utilization of social support.

#### Problem focused coping

All interviewees reported to apply *organizational development* for dealing with experienced work-related stressors, which included to hire new employees, to promote employees to be responsible for leadership tasks, to outsource work tasks or to optimize process structures. One interviewee mentioned that he started to implement changes when realizing that he could not cope with the experienced work-related stressors anymore:

*“And that made me say, okay, something’s not right here, and that’s a certain level of stress that I didn’t really want to have anymore, and that’s when I started thinking about how I could do that within the structure. Within the organizational structure here in the company, I can distribute the tasks among different heads.” [ID 10]*

The problem focused coping approach of *work time arrangement* was mentioned by all interviewees. It involves taking (lunch)breaks, having fix working hours, for example *“from eight to 8 p.m. the focus is on the company, then private” [ID 1]*, being permanently available, also during holidays and weekends, or working on evening or weekends.

Further, all interviewees mentioned behaviors of *work organization* for dealing with experienced work-related stressors. For example, interviewees described to address problems directly when they occur, to plan ahead, to prioritize tasks, to obtain feedback, or to take decisions. One interviewee explained that he is developing pressure on purpose to manage tasks:

*“I function very, very well under pressure. That’s a trick of mine. […] I’m such a last-minute guy […] and it always works. Pressure often makes me better. (I: One could say, you let pressure develop on purpose?) Yes.” [ID 10]*

The *creation of a positive work atmosphere* was mentioned by all interviewees as it enables to improve work performance and wellbeing at work:

*“I had a father who is very impulsive and always goes first and actually made double stress, and distributed the stress to everyone. And I have rather a different tactic. Rather to try to dissolve the stress into a good mood. Because you have to cope with it. There is always a problem behind it. And if you then spread even more pressure, you can’t solve the problem. You have to do exactly the opposite, and that’s what I’m actually trying to do.” [ID 7]*

Interviewees reported that creating a positive work atmosphere is especially important with regard to the work-related stressor of shortage of skilled workers as it prevents, according to their statements, existing employees from leaving the company.

Next to *wage sacrifice* due to existential anxiety *advance training* was described by some interviewees to reduce the experience of work-related stress.

#### Emotional focused coping behaviors

Interviewees mentioned the use of *balancing activities* such as exercising, spending time with family and friends, gardening or cooking as strategies to deal with the experienced work-related stressors. Further, many interviewees reported that their perspective on occurring and handling work-related stressors changed throughout their leadership experience and when reflecting on past work-related actions. This changed perception, which, according to the descriptions of the interviewees, made it easier to deal with work-related stressors, can be summarized as *cognitive restructuring*. For example, one interviewee described that throughout his leadership experience his mindset on work-related problems changed as he recognized that it is normal that problems constantly appear and exist: *“It always burns.” [ID 3]*. In addition, some interviewees reported to have *temper tantrums* when work-related stressors appeared.

#### Utilization of social support

First, utilization of *emotional support* was described by most interviewees as important coping behavior. Emotional support comprises actions of family and friends like distractions, getting rid of worries, for example by talking to life partners, and reinforcement and understanding, for example by having a partner who works in a similar job and therefore understands the experience of work-related stressors and their consequences. Moreover, most interviewees explained to take on *informative support* to cope with the experienced work-related stressors. According to the interviewees informative support is supplied by entrepreneurs, family and friends, and contains exchange on entrepreneurs´ experience, advice on professional issues and on how to deal with work-relates stress. Further, interviewees reported that colleagues, business partners, supervisors and friends and family support them by taking over (work) tasks, backing or maintaining (lunch) breaks and healthy eating habits. These behaviors were categorized as *instrumental support*.

### Resources supporting the coping process

From the interview material, three major categories regarding resources supporting the coping process could be identified: (1) personal resources, (2) social resources, (3) organizational resources.

#### Personal resources

All interviewees described themselves as being *proactive*. This subcategory refers to situations in which the interviewees explained taking personal initiative and were willing to further develop their professional experience, for example by starting a company:

*“I was dissatisfied with my job at the time and I-, I came home in the evening and said, it can’t go on like this. Tomorrow I’m going to start my own business. That’s how it was. And then I went into business for myself the next day. I gave notice and registered my business.” [ID 9]*

*Professional experience* is also named by all interviews as a resource helping to deal with experienced work-related stressors. Acquired skills like hiring employees, conducting negotiations with costumers or guiding employees were described by the interviewees as being developed over time or making it easier to cope with work-related stress.

A further personal resource mentioned by the interviewees was *optimism*. Interviewees reported having a positive attitude towards work and life experiences, which would help to cope with work-related stressors.

In addition, being *communicative* was cited by most interviewees as a factor helping to cope with work-related stressors. Next to feeling comfortable in situations such as contacting potential customers or employees, interviewees explained resolving work-related stressors by talking and getting into conversations with others. Further, one interviewee said:

*“You’re always dealing with other people. And that’s where I draw energy from.” [ID 6]*

Further, *self-confidence* was described by the interviewees as a helpful personal resource. Interviewees reported to feel comfortable and confident in their leadership positions, for example on taking decisions or conducting large business projects with millions of investment sums. One interviewee explained that he feels capable of succeeding in critical moments:

*“I’m a guy who, when it gets critical, when it goes into crisis mode, is pretty strong. So I’m a problem solver, right. So I’m the kind of guy who just gets into top form when things get tricky. You have to be able to do that a bit. Then I already go into the ring and then I also do that, and mostly I am successful with it. And this also gives strength or satisfaction again, so that you don’t fail at it or something. That is also a reason, why I am in this position, right.” [ID 10]*

Next to the description of *endurance* and *personal interest*, interviewees reported that being *reflective* is considered as a helpful resource as it has helped them to initiate and perform changes on business structures, personal attitudes or:

*“What’s also important is that you have to be reflective. You really have to think about it every time, what have I-, where have I made mistakes, or what can I do better, or where are the limits. Because that is already-. There are one or two moments when I think, “Wow, if you had been a little calmer, you would have achieved a better result.“ Because you then botch something again or formulate something incorrectly in the conversation, because you’re simply under pressure. Which is then somehow perceived not as intended. And with the many number of conversations that you have, that can just happen and then you just have to come down at some point.” [ID 7]*

#### Social resources

Social resources helping to deal with work-related stressors can be divided, according to the interview material, into the subcategories *family*, *colleagues* and *entrepreneurs.* Next to family members like life partners, parents, children or other relatives, also colleagues, which include employees, supervisors, team members and professional partners, as well as other entrepreneurs were named by the interviewees as helpful resources in order to cope with the experienced work-related challenges.

#### Organizational resources

Due to the descriptions of the interviewed leaders, *scope of action* could be explored as important organizational resource helping to deal with work-related stressors. For example, the interviewees mentioned that the degrees of freedom reduce the experienced work-related stressors:

*“Of course, the time required is certainly a bit different than when you work purely as an employee, but the degrees of freedom then also give that back as a reward, so that you can just decide what you deal with and what you deal with more intensively.” [ID 1]*

Further, *financial reward* is named by some interviewees as a motivational factor for performing and coping with work-related stress.

### Consequences of work-related stressors

When asked which consequences the experienced work-related stressors have, interviewees listed different aspects, which can be divided into three major categories: (1) consequence of work-related stressors for health, (2) consequence of work-related stressors for work, (3) consequence of work-related stressors for private life.

#### Consequences for health

All interviewees cited experiencing *subjectively perceived psychological effects*. Most interviewees reported that they feel tired and exhausted at the end of working days or working weeks. One interviewee described to feel completely overworked:

*“Yes, it just didn’t work anymore. So completely overworked, completely knocked out. You really felt like shit. So you really felt like shit.” [ID 3]*

Next to exhaustion, many interviewees reported having problems with mental disengagement described as thoughts circling around work and linked to the dissolution of boundaries. Further, many interviewees described that due to the high experienced work stressors sleeping disorders evolved. It was expressed that sleeping problems were occurring when experiencing existential anxiety or before important appointments such as presentations or team events. Other subjectively perceived psychological effects mentioned by the interviewees consisted of irregular and unbalanced nutrition, feeling of being driven, loss of control and teeth grinding.

Major *subjectively somatically perceived effects* described by the interviewees were back complaints, weight gain and digestive problems. Interviewees mentioned that weight gain was experienced as result of irregular and unbalanced nutrition, and digestive problems were mainly experienced in stressful phases. Further subjectively somatically perceived effects reported by the interviewees were visual field loss, colds, high level of stress hormones and painful legs.

#### Consequences for work

A major result of work-related stressors for work mentioned by the interviewees is a *decrease in performance and increase in errors.* For example, it was mentioned that the decrease in performance is experienced as also influencing the employee:

*“Because if I do the five tasks hectically, then mistakes will happen. Or if I have to have a conversation with a project manager and I’m totally flustered and in a hectic state, that’s going to have a negative effect on him.” [ID 2]*

It was mentioned that *rash decisions* are mostly made when time is limited which consequently leads to decisions regretted afterwards:

*“[…] I just notice that, if things are poorly prepared, we go into appointments that are not prepared at all, and then you say things that you might regret a week later.” [ID 5]*

In addition, a *lack of collegial understanding* was described by interviews as a result of different perceptions regarding the individual understanding of work and leadership. Further, *irregular breaks* were mentioned by many interviewees as a result of high work-related stressors.

#### Consequences for private life

Most interviewees indicated *existential anxiety* as a consequence of the experienced work-related stressors. This code includes situations in which interviewees reported that are afraid of economic ruin, unemployment, or not existing as a business in the future anymore.

Several interviewees named the *neglect of private contacts and leisure activities* due to high work load. For example, it was reported that activities such as meeting friends, shopping or household chores are not possible during weekdays, hence these activities shift to the weekends:

*“[…] when I come home from work, I’m exhausted. Then I don’t start anything big now. Then everything automatically shifts to Saturday, everything that you have to do at home on the property. And with shopping times, new clothes, visit someone. Yeah, when should I do all that? Of course, this has an effect. [ID 2]*

Several interviewees also described *reduced family time due to overtime* because of the experienced work-related stressors. Further, interviewees reported about *social incomprehension* by friends regarding their long working hours.

## Discussion

The implementation of effective health-oriented leadership interventions is essential for the promotion of leaders’ and followers’ health and well-being within worksite settings [36]. To create and develop effective interventions, the specific needs of the target group have to be examined first [12]. This is also true for leaders in SMEs in the IT and technological services industry, the specific focus of this study.

The interviews with representatives of this target group revealed – as to be expected – a wide variety of work-related stressors. Next to stressors caused by work organization (e.g. high volumes of work, variety of tasks, working with humans, interruptions, task prioritization) and industry-related work stressors (e.g. economic and deadline pressure) other work-related stressors (e.g. dissolution of boundaries, sitting work posture or lack of appreciation) were named by the interviewees. When comparing these stressors to study results from other occupational settings, similarities can be found. The Stress Report Germany 2019 [37], which analyzed data in diverse occupational settings, also concluded that leaders stated simultaneous supervision of diverse tasks, intense deadline or performance pressure, and interruptions as the most commonly occurring work-related stressors. In addition to these more general stressors, industry-specific stressors could be identified. The interviewees reported to be exposed to a strong (economic) pressure, resulting from extraordinarily quick technological progress in this field, very high product and service demand – due to an exponential growth in digitalization –, and an enormous shortage of skilled workers. Additionally, organization of a balanced workload due to high demands of clients regarding quality and execution time of requested work tasks turned out to be major stressors. The assessed industry-specific stressors expand the results of Gerlmaier who reported work-related stressors like work interruptions, unplanned additional work, time pressure, obstacles to the acquisition of new knowledge, emotional stress, social tensions as well as contradictory work requirements [16]. However, while Gerlmaier primarily included managers and employees in IT enterprises with more than 250 employees [16], our results exclusively relate to leaders working in small and medium-sized IT and technological services enterprises.

In order to cope with the experienced stressors, leaders reported coping strategies that could be categorized into problem focused coping (e.g. organizational development, work time arrangement, work organization, advanced training), emotional focused coping (e.g. balancing activities, cognitive restructuring) and use of social support. Especially the identification of coping strategies addressing changes on the organizational level can be seen as extension of current research findings. So far, mainly coping strategies limited to changes in personal behaviors have been reported [38, 39]. Our data suggest, however, that coping behaviors addressing organizational changes are only performed when negative effects for economic success (e.g. due to high workloads, economic and deadline pressure) or personal health (e.g. exhaustion, visual field loss, digestive problems) are experienced. This was especially true for the enterprise owners with fewer years of leadership experience. Accordingly, structural changes seem to be applied only when personal coping behaviors appear insufficient for the occurring work-related stressors. This is somewhat surprising since superior leaders or company owners as head managers have major power at their disposal. Future research should therefore examine which social, personal and environmental factors are impeding leaders from implementing changes.

Several helpful resources for the coping process were reported by the interviewees which could be classified into organizational, social and personal resources. Next to the important resources of scope for action and social support, which act as effective protective factors for psychological health impairments [40–42], the interviewed leaders reported to take recourse on personal resources. These include personal traits and acquired skills like professional experience, being proactive, communicative or optimistic, and correspond to character traits found in studies on successful managers and entrepreneurs [43–46].

Interestingly, workplace health promotion was neither considered as a coping strategy nor a resource; none of the interviewed leaders talked about performing or wanting to implement workplace health promotion in their enterprises. This observation is especially surprising as one could assume that given industry-related stressors, the margin and scope for decisions on health management should increase. Accordingly, future research definitely needs to examine further which knowledge and understanding of the aim and performance of workplace health promotion exists among leaders in small and medium-sized IT and technological services enterprises. This observation is also important for the development of effective occupational health activities and implies that comprehensive information about the scope, objectives and benefit of workplace health promotion needs to be part of the training curricula.

A further important finding of our study is that leaders, on the one hand, are obviously aware about negative consequences of the reported stressors. On the other hand, they described to apply coping behaviors, e.g. being permanently available or having 12 h working days. These coping strategies, however, can be considered as harmful since they result in having less time for regeneration, family or friends [47]. This behavioral contradiction – to deal with work-related stressors but simultaneously increase the likelihood of health problems by reducing needed time for recovery [48] – is described as self-endangering [49]. Future research should therefore examine underlying mechanisms of self-endangering in leaders. For future health promotion interventions this implies to address this issue and invite the participants to reflect respective behavior as well as their values and attitudes towards their role in particular and their job in general.

As a result of the various experienced work-related stressors, multiple consequences for health, work and private life were reported, including negative health effects on the psychological and somatic level. In addition, negative effects on work quality and leadership performance as well as existential anxiety and work-family conflicts were raised as important issues.

Our results contribute to present research and broaden knowledge on experienced consequences of high specific work demands on leaders in SMEs. Rau et al. concluded that entrepreneurs are more likely exposed to health impairments than the general population [50]. Zimber et al. showed that managers working in different economic sectors experience work-related psychological impairments like fatigue and exhaustion, sleeping problems, insufficient recovery time and having problems to mentally detach from work [51]. Wallis et al. found that team leaders and senior managers reported more work-related challenges and lower satisfaction with their work life balance compared to non-managers [52]. Wach et al. came to the conclusion that entrepreneurs have problems mentally detaching from work [53]. Overall, our study findings regarding the resulting consequences are alarming due to following different reasons, and point out the crucial need for target-oriented health-oriented leadership interventions for leaders in small and medium-sized IT and technological services enterprises: First, the work environment is changing rapidly, increasing the need of IT and related technological services tremendously [21]. A current representative survey documents that 66% of the 851 interviewed business executives and human resource managers expect the shortage of IT professionals to become even worse in the future [54]. Second, exhaustion is a central dimension of burnout [55]. Exhausted leaders are less able to invest time and resources in taking care of their own health [56]. In our study all leaders stated to experience exhaustion due to high work-related stressors. Consequently, first signs of burnout could be recognized in the examined target group. Third, leaders stress is associated with poorer leadership [57]. The meta-analysis performed by Kaluza et al. identified a clear link between leader’s wellbeing and their behavior towards their subordinates [58]. Stein et al. concluded that leaders´ workload creates boundaries for engaging in supportive leadership [59]. Klug et al. showed that leaders´ health awareness correlates positively with follower health and leaders’ health-promoting behaviors [60]. Fourth, due to the COVID-19-pandemic the working environment becomes even more flexible and digitalized, leading to blurring boundaries between professional and private life [61, 62].

In summary, our results contribute to the rare literature of work-related psychosocial stress among leaders in small and medium-sized enterprises. In a previous integrative review only five out of 45 included studies examined work-related psychosocial demands of SME-managers or enterprise owners [63]. Our results also underline the apparent need of occupational health interventions for leaders in SMEs, particularly of those working in IT and technological services. Currently, occupational health interventions for leaders are underrepresented in SMEs [10]. This underrepresentation is remarkable for two main reasons. First, leaders play a key role in enterprises. They influence employees directly through their behavior and communication, and have an indirect influence on employees´ health through the design of working conditions. In addition, as also seen in our interviews, leaders themselves are confronted with various stressors [64]. Leaders exhibit an increased prevalence of psychological impairments [65], have significantly higher depression scores compared to the general population in Germany [66]. Furthermore, leaders act as role models for health behavior and can only authentically represent health promotion if they themselves behave in a health-conscious manner [64]. They influence employees directly through their behavior and communication, and have an indirect influence on employees´ health through the design of working conditions. Therefore, leaders function as connective link between individual health and organizational health promotion and are promoters of occupational health and safety in worksite settings [36]. Second, the target group is large and of economic relevance, as they employ a large body of workforce [8]. Consequently, the development of occupational health interventions for leaders in SMEs in general, and those acting in IT and technological services enterprises, as documented by our results, is highly relevant.

### Limitations

Some limitations must be considered when interpreting the results. First, the study used a convenience sampling scheme and interviewed only ten persons. Even if the study could make meaningful explanations in relation to the study aims, a different or larger sample of individuals might have produced other results. Seond, the subjects of this study were leaders in small and medium-sized IT and technological services enterprises in Germany with max. 250 employees with at least three years of working experience. Results might therefore be valid only for these specific settings. Third, the transactional stress model by Lazarus and Folkmann, and the extension by Bamberg et al. was chosen as theoretical approach, since it provides program planers with important information for development and implementation of occupational health interventions. However, the selection of a different theoretical framework might have led to different results. Fourth, the content-structuring content analysis according to Kuckartz was used for data analysis. It is a content-reductive approach, which provides a thematic overview of the interview material. For the development of effective health-oriented leadership this was considered a suitable approach as it provided clear recommendations for the examined target group. Selection of another analytical approach might have led to different results. Fifth, the interviews were only encoded by one author which may decrease the plausibility of the analysis. Sixth, only one women participated in the study which did not allow to consider gender-related differences. Seventh, interviewees may have answered according to social desirability and might therefore not have displayed all relevant aspects. Finally, it can be assumed that only leaders who currently feel able to cope with stress and are interested in the topic of (occupational) health have agreed to participate in the study. It can be assumed that the full spectrum of work-related stressors, coping behaviors, available resources and emerging consequences could not be captured.

## Conclusion

In conclusion, the identified experienced work-related stressors, applied coping behaviors, utilized resources and emerging consequences underpin the urgent need for the development and performance of health-oriented leadership interventions for leaders in small and medium- sized IT and technological services. The results of this study can be used when designing a target-oriented intervention for the examined target group.

## Electronic supplementary material

Below is the link to the electronic supplementary material.

Supplementary Material 1

Supplementary Material 2

## Data Availability

The datasets generated and/or analyzed during the current study are not publicly available due data protection reasons but are available from the corresponding author on reasonable request.

## References

[1] OECD EU (2018). Health at a glance: Europe 2018: state of Health in the EU cycle.

[2] Meyer M, Wing L, Schenkel A, Meschede M, Badura B, Ducki A, Schröder H, Meyer M (2021). Krankheitsbedingte Fehlzeiten in der deutschen Wirtschaft im Jahr 2020. Fehlzeiten-Report 2021: Betriebliche Prävention stärken – Lehren aus der Pandemie.

[3] Hassard J, Teoh KRH, Visockaite G, Dewe P, Cox T (2018). The cost of work-related stress to society: a systematic review. J Occup Health Psychol.

[4] Gao L, Nguyen P, Dunstan D, Moodie M (2019). Are Office-Based workplace interventions designed to reduce sitting time cost-effective primary Prevention Measures for Cardiovascular Disease? A systematic review and Modelled Economic evaluation. Int J Environ Res Public Health.

[5] Goetzel RZ, Tabrizi M, Henke RM, Benevent R, Brockbank CVS, Stinson K (2014). Estimating the return on investment from a health risk management program offered to small Colorado-based employers. J Occup Environ Med.

[6] Mills PR, Kessler RC, Cooper J, Sullivan S (2007). Impact of a health promotion program on employee health risks and work productivity. Am J Health Promot.

[7] ESNER. What does it tell us about safety and health in Europe’s workplaces?; 2019.

[8] European Union. Unleashing the full potential of European SMEs; 2020.

[9] McCoy K, Stinson K, Scott K, Tenney L, Newman LS (2014). Health promotion in small business: a systematic review of factors influencing adoption and effectiveness of worksite wellness programs. J Occup Environ Med.

[10] Straub R, Schmitt K, Krapf F, Walter UN, Mess F, Arps W (2017). #whatsnext – GESUND ARBEITEN IN DER DIGITALEN ARBEITSWELT.

[11] Badura B, Faller G (2017). Die zentrale Bedeutung der psychischen Gesundheit: Für eine Kultur der Achtsamkeit und des Sozialvermögens. Lehrbuch Betriebliche Gesundheitsförderung.

[12] Bartholomew Eldredge LK, Markham CM, Ruiter RAC, Fernández ME, Kok G, Parcel GS (2016). Planning health promotion programs: an intervention mapping approach.

[13] Ducki A, Badura B, Ducki A, Schröder H (2019). Digitale Transformation - von gesundheitsschädigenden Effekten zur gesundheitsförderlichen Gestaltung. Fehlzeiten-Report 2019: Digitalisierung - gesundes Arbeiten ermöglichen.

[14] Destatis. Statistisches Jahrbuch: Deutschland und Internationales; 2019.

[15] Destatis. Unternehmen, tätige Personen, Umsatz und Investitionen in der IKT-Branche. 2021. https://www.destatis.de/DE/Themen/Branchen-Unternehmen/Unternehmen/IKT-in-Unternehmen-IKT-Branche/Tabellen/iktb-03-unternehmen-tetige-umsatz-investitionen.html. Accessed 2 Dec 2021.

[16] Gerlmaier A, Gerlmaier A, Latniak E (2011). Stress und Burnout bei IT-Fachleuten - auf der Suche nach Ursachen. Burnout in der IT-Branche: Ursachen und betriebliche Prävention.

[17] Gerlmaier A, Gerlmaier A, Latniak E (2011). Psychische Erschöpfung in der IT-Arbeit - Welche Rolle spielt die individuelle Arbeits- und Lebensphase. Burnout in der IT-Branche: Ursachen und betriebliche Prävention.

[18] Boes A, Kämpf T, Trinks K. Gesundheit am seidenen Faden: zur Gesundheits- und Belastungssituation in der IT-Industrie. In: ver.di - Vereinte Dienstleistungsgewerkschaft, editor. Hochseilakt - Leben und Arbeiten in der IT-Branche. Berlin; 2009. p. 53–64.

[19] Jung E (2013). Work stress and burnout: the mediating role of Mood Regulation among Information Technology Professionals. J Workplace Behav Health.

[20] Maudgalya T, Wallace S, Daraiseh N, Salem S (2006). Workplace stress factors and ‘burnout’ among information technology professionals: a systematic review. Theoretical Issues in Ergonomics Science.

[21] Hofmann JC, Badura B, Ducki A, Schröder H, Meyer M (2021). Arbeit in Zeiten von Gesundheitskrisen – Veränderungen in der Corona-Arbeitswelt und danach. Fehlzeiten-Report 2021: Betriebliche Prävention stärken – Lehren aus der Pandemie.

[22] Rudolph CW, Allan B, Clark M, Hertel G, Hirschi A, Kunze F (2021). Pandemics: implications for research and practice in industrial and organizational psychology. Ind Organ Psychol.

[23] Röhrborn D. Der IT-Mittelstand in Deutschland. 2017. https://www.bitkom-research.de/en/system/files?file=document/170111_Mittelstandsbericht_PK_05.pdf. Accessed 16 Apr 2022.

[24] Lazarus RS, Folkman S, Stress. Appraisal, and Coping: Springer Publishing Company; 1984.

[25] Bamberg E, Busch C, Ducki A (2003). Stress- und ressourcenmanagement: Strategien und Methoden für die neue Arbeitswelt.

[26] Ernst G, Franke A, Franzkowiak P. Stress und Stressbewältigung. Bundeszentrale für gesundheitliche Aufklärung (BZgA); 2022.

[27] Kauffeld S, Ochmann A, Hoppe D, Kauffeld S (2019). Arbeit und Gesundheit. Arbeits-, Organisations- und Personalpsychologie für bachelor: Mit 42 Tabellen.

[28] Demerouti E, Bakker AB, Nachreiner F, Schaufeli WB (2001). The job demands-resources model of burnout. J Appl Psychol.

[29] Zapf D, Semmer NK, Schuler H (2004). Stress und Gesundheit in Organisationen. Enzyklopädie der Psychologie.

[30] Lamnek S, Krell C (2016). Qualitative Sozialforschung: Mit Online-Materialien.

[31] Witzel A. The Problem-centered Interview 2000. 10.17169/fqs-1.1.1132.

[32] Tong A, Sainsbury P, Craig J (2007). Consolidated criteria for reporting qualitative research (COREQ): a 32-item checklist for interviews and focus groups. Int J Qual Health Care.

[33] Creswell JW (2013). Qualitative inquiry and research design: choosing among five approaches. Los Angeles, Calif.

[34] Claussen J, Jankowski D, Dawid F, Aufnehmen (2020). Abtippen, Analysieren: Wegweiser zur Durchführung von interview und transkription.

[35] Kuckartz U (2018). Qualitative inhaltsanalyse. Methoden, Praxis, Computerunterstützung.

[36] Dannheim I, Ludwig-Walz H, Buyken AE, Grimm V, Kroke A (2021). Effectiveness of health-oriented leadership interventions for improving health and wellbeing of employees: a systematic review. J Public Health (Berl).

[37] Bundesanstalt für Arbeitsschutz und Arbeitsmedizin (BAuA). Stressreport Deutschland 2019: Bundesanstalt für Arbeitsschutz und Arbeitsmedizin (BAuA); 2020.

[38] Gerlmaier A, Latniak E (2011). Burnout in der IT-Branche: Ursachen und betriebliche Prävention.

[39] Page D (2013). Managers coping in further educational colleges. J Mgmt Dev.

[40] Schwarzer R (2004). Psychologie des Gesundheitsverhaltens: Einführung in die gesundheitspsychologie.

[41] Zijlstra FRH, Sonnentag S (2006). After work is done: psychological perspectives on recovery from work. Eur J Work Organizational Psychol.

[42] Niemann D (2019). Die Rolle des Partners und der Partnerin bei der Bewältigung arbeitsbedingter Belastungen.

[43] Lounsbury JW, Sundstrom ED, Gibson LW, Loveland JM, Drost AW (2016). Core personality traits of managers. J Managerial Psych.

[44] López-Núñez MI, Rubio-Valdehita S, Díaz-Ramiro EM (2022). The role of individual variables as antecedents of entrepreneurship processes: emotional intelligence and self-efficacy. Front Psychol.

[45] Zhao H, Seibert SE (2006). The big five personality dimensions and entrepreneurial status: a meta-analytical review. J Appl Psychol.

[46] Kanning UP, Kanning UP (2019). Stabile Eigenschaften der Manager. Managementfehler und Managerscheitern.

[47] Rau R, Badura B, Ducki A, Schröder H, Klose J, Meyer M (2012). Erholung als Indikator für gesundheitsförderlich gestaltete Arbeit. Fehlzeiten-Report 2012: Gesundheit in der flexiblen Arbeitswelt: Chancen nutzen - Risiken minimieren.

[48] Dettmers J, Deci N, Baeriswyl S, Berset M, Krause A, Wiencke M, Cacace M, Fischer S (2016). Self-endangering work behavior. Healthy at work.

[49] Peters K. Indirekte Steuerung und interessierte Selbstgefährdung. Eine 180-Grad-Wende bei der betrieblichen Gesundheitsförderung. In: Kratzer N, Dunkel W, Becker K, Hinrichs S, editors. Arbeit und Gesundheit im Konflikt: Nomos; 2011. p. 105–122. 10.5771/9783845271231-105.

[50] Rau R, Hoffmann K, Metz U, Richter PG, Rösler U, Stephan U. Gesundheitsrisiken bei Unternehmern. Zeitschrift für Arbeits- und Organisationspsychologie A&O. 2008;52:115–25. 10.1026/0932-4089.52.3.115.

[51] Zimber A, Hentrich S, Meyer-Lindenberg A. „Dass ich das ändern muss, damit ich nicht irgendwann kollabiere ?“. [Mental Health Risks Among Managers - Results of a Qualitative Study and Implications for Stress Prevention]. Psychiatr Prax. 2018;45:30–7. 10.1055/s-0042-122163. 10.1055/s-0042-122163 28371956

[52] Wallis A, Robertson J, Bloore RA, Jose PE (2021). Differences and similarities between leaders and nonleaders on psychological distress, well-being, and challenges at work. Consulting Psychol Journal: Pract Res.

[53] Wach D, Stephan U, Weinberger E, Wegge J (2021). Entrepreneurs’ stressors and well-being: a recovery perspective and diary study. J Bus Ventur.

[54] Pauly B. IT-Fachkräftelücke wird größer: 96.000 offene Jobs. 2022. https://www.bitkom.org/Presse/Presseinformation/IT-Fachkraefteluecke-wird-groesser. Accessed 16 Apr 2022.

[55] Maslach C, Schaufeli WB, Leiter MP (2001). Job burnout. Annu Rev Psychol.

[56] Köppe C, Schütz A (2019). Healthy leaders: core self-evaluations affect leaders’ Health Behavior through reduced exhaustion. Front Psychol.

[57] Harms PD, Credé M, Tynan M, Leon M, Jeung W (2017). Leadership and stress: a meta-analytic review. Leadersh Q.

[58] Kaluza AJ, Boer D, Buengeler C, van Dick R (2020). Leadership behaviour and leader self-reported well-being: a review, integration and meta-analytic examination. Work Stress.

[59] Stein M, Vincent-Höper S, Gregersen S (2020). Why busy leaders may have exhausted followers: a multilevel perspective on supportive leadership. LODJ.

[60] Klug K, Felfe J, Krick A (2019). Caring for oneself or for others? How consistent and inconsistent profiles of health-oriented Leadership are related to follower strain and health. Front Psychol.

[61] Carnevale JB, Hatak I (2020). Employee adjustment and well-being in the era of COVID-19: implications for human resource management. J Bus Res.

[62] Zeike S, Bradbury K, Lindert L, Pfaff H (2019). Digital Leadership Skills and Associations with Psychological Well-Being. Int J Environ Res Public Health.

[63] Schreibauer EC, Hippler M, Burgess S, Rieger MA, Rind E (2020). Work-related psychosocial stress in small and Medium-Sized Enterprises: an integrative review. Int J Environ Res Public Health.

[64] Franke F, Ducki A, Felfe J, Felfe J (2015). Gesundheitsförderliche Führung. Trends der psychologischen Führungsforschung.

[65] Zimber A, Hentrich S, Bockhoff K, Wissing C, Petermann F (2015). Wie stark sind Führungskräfte psychisch gefährdet?. Z für Gesundheitspsychologie.

[66] Hentrich S, Zimber A, Sosnowsky-Waschek N, Gregersen S, Petermann F (2017). The role of core self-evaluations in explaining Depression and Work Engagement among managers. Curr Psychol.

