# Supplementary material for: Work-related stressors and coping behaviors among leaders in small and medium-sized IT and technological services enterprises
Source: BMC Public Health. 2023 Apr 14;23:700. doi: 10.1186/s12889-023-15581-3 (PMC10103039; doi:10.1186/s12889-023-15581-3)
Supplement: Supplementary file 2 — Supplementary Material 2 [file 12889_2023_15581_MOESM2_ESM.pdf]

Appendix_additional file 2

Work -related stressors and coping behavior among leaders in small and medium-sized IT and technological services enterprises

Table 1: Summary of the identified work-related stressors including selecting 2
Table 2: Summary of the identified coping strategies 4
Table 3: Summary of the identified resources promoting the coping process 12
Table 4: Summary of the identified consequences of the experienced work-related stressors 14

Corresponding author
Indra Dannheim | indra.dannheim@oe.hs-fulda.de

Regional Innovative Centre of Health and Quality of Live Fulda (RIGL), Fulda University of Applied Sciences, Fulda, Germany || Department of Nutritional, Food and Consumer Sciences, Fulda University of Applied Sciences, Fulda, Germany

Table 1: Summary of the identified work-related stressors including selecting

Major category	Subcategories	Selective quotes
**Work-related stressors caused by work organization**	High volumes of work	“So as I said, the first years-, even now I work a lot. So under 60 hours there is actually little.” [ID 7]<br>“But of course it's just a lot of work.” [ID 3]
	Working with humans	“I: What would you say is the biggest challenge for you as a leader?<br>B: The 30 employees. Yes, well, of course I always say that for fun, but it's just really like that, you're just a bit of a psychologist.” [ID 9]
	Variety of tasks	B: The biggest challenge is certainly to accommodate a number of things, the actual business, the actual daily project work, of course, the superordinate and also, to have notion of the projects, and also to acquire projects. [ID 10]
	Acceptance of responsibility	“As a leader, you are an escalation manager, as the saying goes. Basically, you have to deal with things that don't work out most of the time. Yes. And that takes its toll on you. And you have to make sure, in some way, that you can still sleep.” [ID 6]
	Interruptions	“For me, it's just that I have ten pieces of paper with ten tasks on my desk. Then I have to see how I can prioritize them. When I've got that reasonably under control, the phone rings or someone comes by or an e-mail arrives saying that something has to be done quickly. I have to organize that.” [ID 2]
	Delegating work	“… you can't do everything. And that's sometimes hard for me. Goes a little bit on helper syndrome. So if I notice that a coworker can't do it or something, I'm already straining, "Do you need assistance?" And that might be a mistake sometimes. And these are issues where I actually try to work on myself, so that people also-. Sometimes you have to do it without, you also have to bite your way through. And maybe I come too early with the helping hand.” [ID 7]
	Task prioritization	“Well, one stress factor is managing different tasks that have the same high priority. If I know I have to do five things that are important, I have to do them today and I have a meeting from ten to twelve. And then from 2 p.m. to 4:30 p.m. and I want to finish work at six o'clock and not at eight. This stresses me out.” [ID 2]
**Business related work stressors**	Economic pressure	„Well, the biggest challenge, that took several years. You have to see, I took over a small company that had a very bad reputation. It also reported negative figures in not a small amount. In other words, the difficult thing was to generate customers in the first place. And of course, that was mainly done through price. So we really tried to gain their trust. To make people realize: "Okay. He can do that. But this is still such a young guy, under 30, and he's trying himself out there." And that, quite honestly, was only possible through the price. [ID 7]
	Organization of a balanced workload	“Ah, I still have something that stresses me out. (I: Yes.) And that is, of course, too much work or too little work. Now in the company. Because with me it's the whole thing. I have to make sure that all the people have something to do. There are certain times when there is too little work, when I have to ask myself, "What do you want to do with the people? Where you have to see where you can get something. And when there is too

		much work, it is of course difficult. Now a customer wants to have something done by us, so of course I'm happy. But if I don't have anyone who can do it in the same quality? And then there are, I see problems coming, that is of course also a stress factor.” [ID 2]
	Technological progress	“And there are a lot of new technologies. So we have to see how it develops further and we must not lose touch, and we have to position ourselves well.” [ID 5]
	Deadline pressure	“Or whatever the issue is, we always have a lot of pressure to deliver, deadline pressure, as I just said, when we get asked some project: Can you do it in four weeks? and we say yes. Then that's pretty bang on.” [ID 10]
	Shortage of skilled workers	“… of course, because what drives us the most in the IT industry is the shortage of skilled workers.” [ID 9]
**Other work-related stressors**	Dissolution of boundaries	But that [the leadership position] also changes the character of oneself, you must not forget that either. Because it's also hard to get out of this leadership role. That means that you also apply this at home with friends and children. That's automatically the case. I always have discussions with my wife when we talk about work topics. She then says, "Yes, now you're coming with your executive topics. This is not a staff meeting here." I mean, you don´t want to turn a private conversation into a staff meeting, but you just notice that the leadership position is in you. [ID 7]
	Sitting work posture	“What's stressing me out at the moment, I'd like to say, is this constant sitting at my desk. Because I'm either looking at the screen or these stupid video conferences all the time.” [ID 2]
	Videoconferences	“Looking into the camera, sounds stupid, but somehow it also stresses you out. You see, the others see you. I haven't quite figured out yet why that is. Here, when you're sitting across from each other, you don't think about it. That's funny.” [ID 2]
	Talking	“But my job means that I'm constantly talking to people every day. And I find that exhausting. So when I have days where I really have one appointment after another, where I'm always in contact, I'm totally exhausted in the evening.” [ID 3]
	Lack of appreciation	You always get a lot of criticism. And that's actually what you always get. So that a customer says, "That was really great." That happens very, very rarely. Because, unfortunately, this is rarely communicated in business. And if something doesn't go one hundred percent, that's always put on the plate. "And here, now that wasn't so great and that wasn't so great." [ID 7]

Table 2: Summary of the identified coping strategies

**Major category**	**Subcategories**	**Dimensions**	**Selective quotes**
**Problem focused coping**	Organizational development	Process structuring	“We have invested a lot of time over the last year to make that leap from creating a process that allows everyone to spend at least 80 to 90 percent of their time doing the tasks to our standards and error-free. So that the input required is reduced. [ID 3]<br>“And that made me say, okay, something's not right here, and that's a certain level of stress that I didn't really want to have anymore, and that's when I started thinking about how I could do that within the structure. Within the organizational structure here in the company, I can distribute the tasks among different heads.” [ID 9]
		Employee empowerment	“Of course, this requires that I have good people in my projects who can then, under certain circumstances, also make their own decisions. And that is, let's say, the main goal of mine or of my daily work, that my people in the background are educated very early on to make their own decisions, and that they are also allowed to do so in part, that I bring them into a certain leadership role in my projects, that they can also represent me in case of doubt when I am generally active for the office.” [ID 10]<br>“We also formed team leaders to pull in a bit of second-tier management as well.” [ID 1]
		Hiring employees	“We have hired six people here.” [ID 8]<br>And that's why there is now Mrs. [name], because we have realized that we can no longer manage to look after them [employees] ourselves. That's why we now have [name] to take care of them.” [ID 7]

		Outsourcing	"We have outsourced almost everything." [ID 1]
	Work time arrangement	Fix working hours	"I have defined for myself, the day has two halves. The first twelve hours and the second twelve hours. In the first twelve hours, I deal with company issues. In the second twelve hours, I deal with my own private issues. That means from eight to eight. Or from eight to 8 p.m. the focus is on the company, then private. [ID 3]
		Expansion of working hours	"Good, and then there are things that I can't do in my normal working hours. Where I then just drive here on Saturdays or Sundays for two hours, three hours, four hours. And to simply get rid of it, because it would otherwise be left lying around. Yes, that's also part of it." [ID 2]
		Permeant availability	"I sometimes respond on vacation. Of course, this is not during the day on the beach, but in the evening, when the others are asleep, or so, then I will already also, to be able to act quickly and flexibly, I can then already answer a few things. That also has to do with the fact that I don't have to work off the wave, or the bow wave, after the vacation, and I've already done the most important things. But of course, as a manager in a company like this, which is global and operates quickly, I also have to do this in the evenings and sometimes at the weekend. So you are also available and can also be reached by phone at 9 p.m. in the evening and then you just go there. Yes. It doesn't matter how much you do immediately, but in our case, a manager is also, I would say, I am available." [ID 10]
		(Lunch) break	"Short breaks are good for me." [ID 3]<br>"(I: And what about your break, lunch break?) Very

			consistently for many years. One of the most important breaks in the day, yes. At least an hour and a half. Would say over the last ten years quite consistently, yes." [ID 1]
	Work organization	Structured work planning	"So we just plan a lot. We have then just. We have our liquidity planning, which virtually goes a whole year in preview and says, when do we need how much money? How many costs do we have? At which points will we have which revenues and which expenses? This allows us to pre-cast extremely well and say what will happen. That takes an extreme amount of stress out of there. Or gives you enough stress early on to make sure that it's not going to be a problem." [ID 3]
		Addressing problems directly	"So if a problem arises somewhere, I want to have it resolved immediately. That means I communicate with the respective people immediately and try to eliminate the problem as quickly as possible. [ID 9]
		Taking decisions	"You must also dare to make a decision if you think it is not possible at the moment. Have variables at hand that you can or must determine in order to make the decision." [ID 1]
		Prioritizing tasks	"I also do the staffing and, let's say, the trick is to always work according to importance." [ID 10]
		Letting pressure develop	"I function very, very well under pressure. That's a trick of mine. […] I'm such a last-minute guy […] and it always works. Pressure often makes me better. (I: One could say, you let pressure develop on purpose?) Yes." [ID 10]
		Obtaining feedback	"I have already received a lot of feedback on what I would perhaps do differently or what [name] would do. We also always ask the customers. It's very

			important to us that we improve.” [ID 8]
	Creation of a positive work atmosphere		“Accordingly, you should also take care of your employees, so that we don't end up in such a fluctuation rate all the time. That means it's important that the people you have, and we have an extremely good, homogeneous team right now-. We have a lot of fun at work. And it would be pretty stupid not to work on making everyone feel comfortable here. Accordingly, that's not entirely altruistic, of course, yes.” [ID 9]
	Advanced training		I also attended many seminars, looked at things, had things explained to me. [ID 1]
	Wage sacrifice		“And that was, quite honestly, only about the price. So one has also waived a lot of money, simply to look-. One had also now-, one could present nothing. So there were a few months where I didn't get a salary myself. Our employees also often had to wait two or three weeks for their salaries. That wasn't quite so easy.” [ID 7]
**Emotional focused coping**	Balancing activities	Exercise	“And in the evening I also make sure that I move again. So at least run a round, that is, walk. At the moment I jog once a week. Also only 20 minutes, but that's better than nothing. And otherwise I had always played soccer. That's starting again now. So the physical activity helps me.” [ID 2]
		Spending time with family and friends	“When I have a long day, I'm home at 7 p.m. and then my colleague calls me on the sports field, "Here, can you take a look, this and that aren't working, we need to buy something new. Then I take my bike and go to the sports field. I'm happy to see people and make small talk and then I take care of it and

			then I go home again. But that's not stress for me." [ID 4]
		Vacation	"I allow myself a vacation three times a year. Certainly not always three weeks, but so that I have a clear distance." [ID 1]
		Gardening	"We have a big yard around the house. (...). Others go to the stadium, I just do this. For me it's a relaxation exercise, or I enjoy it and have fun doing it, and that's it. Well sure I could get a gardener, but you like-. I probably don't like to be as efficient as a professional gardener, that's for sure. But that's not the point. It's about the fact that this is a balance for me and such a switch off." [ID 10]
		Cooking	"Then I usually do the cooking. Because I like to cook. That's where I can let off steam a little bit." [ID 7]
	Cognitive restructuring		"So before it was always an oh God and I have to do that too, and now we have to do that too. And then you worked through all the things that were burning, piece by piece. So there are always things that burn. And I just read a book about Jung von Matt. (…) They have 1,000 employees, so it's really a huge company. (…) And they also have house rules. Similar to the one that hangs on the toilet, for example. And the first house rule is, although it's been around for 30 years, it always burns. And that sums it up pretty well as an entrepreneur. If I as an entrepreneur have the feeling that there is nothing to do or there is nothing urgent, then I simply haven't looked. There is no such state as the company is done. There are always issues somewhere that are not going well or where there are problems. And the only difference I think is the question, am I focusing

			on the problems? So a year and a half ago it was very often, oh God and the problem and the problem and the problem and the problem. And then you jumped from one problem to the next and did them that way. And that was also good. You had to get those done, too. But didn't take the step after that to make sure they didn't come back again.” [ID 3]
	Temper tantrums		“But of course there are also moments when even I lose my temper or don't behave the way I would like to.” [ID 7]
**Utilization of social support**	Emotional support	Getting rid of worries	But we already talk a lot about work in private. Me, my wife, that's important to me, to her too. Because she always wants to know how things are going, and how was your day? And she also knows the one or two problems. And sometimes, when you have a difficult appointment or-, it's just really about a new project, of course, she also asks: “What do you think?” Yes, so I'll do that. [ID 8]
		Distraction	“My children, for example, helped me a lot. In the past, the issue was often that you came home and were very stressed. And you couldn't get any more peace and quiet because you were still thinking about it. And until you can then fall asleep and at some point-, that doesn't make the whole thing any better. And now with the children it's different. You drive into the parking lot, open the door and then they come running towards you and "Daddy, Daddy, Daddy." And then that's gone from your mind, too. So you really have to say that makes it easier. [ID 7]
		Understanding and reinforcement	“So family, partner, some community, so you need something where you have a backing. For me, it's the family.” [ID 1]

			"I have a wife who understands this and who also knows where I work and how I work. And she has a similar job to mine now and also has personnel responsibility for ten employees. So I'd say she already knows what that means." [ID 4]
	Informative support	Exchange of entrepreneurs' experience	"Well, I had already been told before, I had talked a lot with other entrepreneurs, they had already told me that you need five years to build up a company. And the first three years are very, very hard. And I can also reflect that one-to-one." [ID 7]
		Advice on professional issues	"I have a fellow student who works in sales. I can talk to him about sales topics. Yes, so there are one or two people where you can get an advice." [ID 2]
		Advice on dealing with work-related stress	Once a very good buddy said to me (…). He once said to me, he was a big boss, he said, [name], you go to your boss and ask him: "What am I am worth?" I can't do that! He said: "Yes, do it, you go there and ask about your value, you need a raise of salary." So I went, and I've always done bigger projects. And my old boss then: "No, for a salary increase? And then I went back to my friend and told him: "Yes, he said no.", "You see, now you know what you're worth, so don't worry so much." [ID 8]
	Instrumental support	Taking over (work) tasks	"We are a large group, so it's easy to distribute the work and you can rely on the others (I: Yes, yes.) That's quite nice." [ID 5]
		Backing	"Of course it's nice to have a business partner you can ask questions. Many, of course, important decisions or large purchases, now an IT server for 100,000 euros, are discussed in pairs. That's quite clear. But it's also good to have someone there when you're not

			completely alone. You can also ask someone who is involved in this situation. An employee or perhaps a manager who has moved to another level, who understands it differently. That's actually quite good. It fits." [ID 8]
		Maintaining (lunch) breaks and eat healthy	"Well, my wife has a Pilates studio. And I've been married for over 30 years. And my wife has been buying only organic quality (…) And I have also been strongly influenced by it over the years. I still like to eat sweets. That's a bit of an issue. But I try not to overdo it. So nutrition is an important topic." [ID 6]<br>"(…) one of the managing directors is a bit older, he places importance on maintaining lunch breaks. (…) Even when we are in the project, he says, from 12 o'clock to 1 o'clock, of course also due to his own own interest he goes to eat (I: Yes, yes.), well, we therefore keep it that way.“ [ID 5]

Table 3: Summary of the identified resources supporting the coping process

Major category	Subcategories	Selective quotes
**Personal resources**	Proactive	"I was dissatisfied with my job at the time and with my job at the time and I-, I came home in the evening and said, it can't go on like this. Tomorrow I'm going to start my own business. That's how it was. And then I went into business for myself the next day. I gave notice and registered my business." [ID 9]
	Professional experience	"Yes, of course, well, you also grow into it, and you also become more confident in your appearance, towards employees, towards customers, partners, you do notice that. In the past, you always thought to yourself, yes, I can say this and that now, is it really the right thing to say, there is someone sitting across from me who is 30 years older, what do I actually want to tell him (I: Yes.). But when you realize, okay, maybe you're not so stupid after all, and what you say works in most cases, then of course you gain confidence and become a bit more self-assured, and yes, I think that's what I've noticed." [ID 5]
	Optimism	"So this one, the glass is half full not half empty. This look is just important." [ID 6]
	Communicative	"Every day-, well, I talk to many people, different people, every day now. For me, it's not difficult." [ID 8]<br>"Then I am in the first line, the one who just talks to someone first. Yes. You're always dealing with other people. And that's where I draw energy from." [ID 6]
	Self-confidence	"So you really have to have strong endurance and also great self-confidence. Because sometimes that's not easy." [ID 7]<br>"I'm a guy who, when it gets critical, when it goes into crisis mode, is pretty strong. So I'm a problem solver, right. So I'm the kind of guy who just gets into top form when things get tricky. You have to be able to do that a bit. Then I already go into the ring and then I also do that, and mostly I am successful with it. And that then also gives strength or satisfaction again, so that you don't fail at it or something. That is also a reason, why I am in this position, right." [ID 10]
	Reflective	"What's also important is that you have to be reflective. You really have to think about it every time, what have I-, where have I made mistakes, or what can I do better, or where are the limits. Because that is already-. There are one or two moments when I think to myself, "Wow, if you had been a little calmer, you would have achieved a better result." Because you then botch something again or formulate something incorrectly in the conversation, because you're simply under pressure. Which is then somehow received differently, as you want. And with the many number of conversations that you have, that can just happen and then you just have to go down at some point." [ID 7]
	Endurance	"So you really have to have strong endurance and also great self-confidence. Because sometimes that's not easy." [ID 7]
	Personal interest	"Of course, you still read some things, some IT newspapers, or something. (I: Yes, yes), but you just do that on the side. But I don't see that as work either. I'm interested in it (I: Yes, yes), so from that point of view it's not a burden." [ID 5]
**Social resources**	Family	"(I: And what helps you in your personal and professional life to better cope with the stresses and challenges that you have?) I: My daughter. Yes. She does, there's quite a bit. Then you always realize, none of that really matters. (I: Yes. Takes you away then.) I: Completely, yes. So family is

		definitely, yes, that's definitely what grounds you a bit and then shows you again that it's not all that important." [ID 9]
	Colleagues	"Which people support me? (I: Yes.) Yes, of course the team here. [ID 5]
	Entrepreneurs	"And then we actually kind of looked for coaches or something similar who could support you. And who is there? What can you do? And then we asked around. And then a year and a half ago, I think, we went into a mastermind like that or we were asked whether we wanted to go to a mastermind by entrepreneur friends of ours. And then started to exchange ideas. And then we started to look at how they do it. [ID 3]
**Organizational resources**	Scope for action	"Of course, the time required is certainly a bit different than when you work purely as an employee, but the degrees of freedom then also give that back as a reward, so that you can just decide what you deal with and what you deal with more intensively." [ID 1]<br>"Yes, I enjoy the fact that I can design as I like. I'm relatively free in that respect. I have a lot of freedom. And I can say: "We can work in that direction and we can work in that direction. I can give impulses for the entire company, which is also gratefully received. Because I can't decide everything, but I can say, "Here in this direction, we should do something." Often it is done, sometimes it is not done, in the sense that I would like it to be done. But that's what I find, that's what I enjoy." [ID 2]
	Financial reward	"And money is also a motivating factor for me. (I: Legitimate.) When I was choosing a course of study, I also looked at where I could earn money. I also looked at archaeology, but that is also a shaky candidate. And biology, you rarely get rich with it. And as a manager, it's up to me how much money I earn in the end. And that's also one thing where I say, "I enjoy it when you see what's in the account at the end of the year." And then I also like to do that." [ID 2]

Table 4: Summary of the identified consequences of the experienced work-related stressors

Major category	Subcategories	Dimensions	Selective quotes
**Consequences for health**	Subjectively perceived psychological effects	Exhaustion	“Yes, it just didn't work anymore. So completely overworked, completely knocked out. You really felt like shit. So you really felt like shit.” [ID 3]
		Thoughts circling around the work	“But basically, your head is always in the company 24 hours a day, no matter what you're doing.” [ID 7]
		Sleeping disorders	“You can't sleep. I got up part of the night, took a piece of paper and wrote down things that came to my mind. I read a book at four in the morning because I couldn't sleep. That's how it is. In phases like that, a lot is demanded of you.” [ID 1]
		Irregular and unbalanced nutrition	“And nutrition is also a problem. You can take care of it yourself. I think I'm not so badly briefed by my wife. But you're on the road a lot. You often don't have any options at all. So when you're driving on the highway, McDonald's, Lacys, Burger King, everything that's there. You can also order a salad. But then you're also annoyed and stressed and you say, "Three days in a row a salad, now you need a treat again." And then it's also irregular. It's not like I can say, "I'm going to lunch every day at noon." I can't. So sometimes I've noticed, then around 4 p.m., "Oh, crap, didn't have breakfast, didn't have lunch. Now you need something slowly." So that's really hard to control and I don't know a definitive solution, I really have to say.” [ID 7]
		Feeling of being driven	“And I realized that I always wanted to do everything really quickly. So I even jumped up and down the stairs because I thought, "Ah, you can save time there."” [ID 1]
		Teeth grinding	“And I also notice that the subject of stress is simply starting to affect my health. For example, it just starts at the dentist with the grinding. So I grind and that is also an issue that is apparently triggered by stress.” [ID 7]
	Subjectively somatically perceived effects	Back complaints	“Yes, physically it's like this, I have back, 32. (laughs) It's embarrassing, but that's the way it is.” [ID 8]
		Weight gain	“But as I said, with the kilos that I have gained, I have to slowly pay attention to it. But I would rather be the person who then does more sports and just already tries to pay attention to the diet. But when you're running from appointment to appointment, you're really dependent on what's around you. Unfortunately, that's the way it is.” [ID 7]

Table 4: Summary of the identified consequences of the experienced work-related stressors

		Digestive problems	"Typical digestive problems or similar. You can see that quite well. In retrospect, one can already say that in such phases of extreme tension, exactly these problems also come, yes." [ID 1]
		Visual field loss	"In fact, when it was that bad, I was also in the hospital for three weeks because of a suspected stroke. So visual field loss. So it was very severe. Really very, very severe." [ID 3]
		Colds	"What I've noticed is, since I'm a manager here now and I'm in the office, I have more colds. That wasn't the case before." [ID 8]
		High stress hormones	"I also had a blood test once, and they said, "Well, the stress hormones are very high." And they would probably also hit the liver a bit slowly." [ID 7]
		Painful legs	"The body just wants to move. And I have to do that in the evening, otherwise-. I haven't done that either, or sometimes it doesn't work. Now here, when I'm on the road for two days on business. Then I have dinner, then we are really two days only in the car or on the chair. And then I notice physically that my legs hurt and so on. So I notice that when that's missing." [ID 1]
**Consequences for work**	Decrease in performance and increase in errors		"Because if I do the five tasks hectically, then mistakes will happen. Or if I have to have a conversation with a project manager and I'm totally flustered and in a hectic state, that's going to have a negative effect on him." [ID 2]
	Rash decisions		"[…] I just notice that, things are poorly prepared, we go into appointments that are not prepared at all, and then you say things that you might regret a week later." [ID 5].
	Lack of collegial understanding		"And that others don't necessarily like the way it's handled, you can say that, too. I would be lying if it were otherwise." [ID 4]
	Irregular breaks		"And sometimes there is so much that it is not possible. Then I just don't take a break or I take it an hour later." [ID 1]
**Consequences for private life**	Existential anxiety		"But otherwise, the biggest problem is more the psychological pressure. If you have existential fears. If you're worried that what you're doing isn't right. That's an enormous burden." [ID 1]
	Neglect of private contacts and leisure activities		"(…) when I come home from work, I'm exhausted. Then I don't start anything big. Then everything automatically shifts to Saturday, everything that you have to do at home on the property. And with shopping times, new clothes, visit someone. Yeah, when should I do all that? Of course, this has an effect. [ID 2]<br>"As I said, you also want to do sports, you want to have a friendship circle and

			something. And you notice that this is getting smaller and smaller.” [ID 7]
	Reduced family time due to overtime		“… of course, each of us [interviewee and life partner] works in the evening. Every day. On the weekends, we also do something. There's something that gets done. It has a lot of time.” [ID 3]
	Social incomprehension		“The other day I was talking to a colleague at work, and he said, "Well, I have to be honest, I don't have as much free time as you do, or as much working time as you put into it." [ID 2]
